# FedRAD: Heterogeneous Federated Learning via Relational Adaptive Distillation

**DOI:** 10.3390/s23146518

**Published:** 2023-07-19

**Authors:** Jianwu Tang, Xuefeng Ding, Dasha Hu, Bing Guo, Yuncheng Shen, Pan Ma, Yuming Jiang

**Affiliations:** 1 College of Computer Science, Sichuan University, Chengdu 610065, China; tangjw@stu.scu.edu.cn (J.T.); dingxf@scu.edu.cn (X.D.); hudasha@scu.edu.cn (D.H.); guobing@scu.edu.cn (B.G.); 2020223045164@stu.scu.edu.cn (P.M.); 2 Big Data Analysis and Fusion Application Technology Engineering Laboratory of Sichuan Province, Chengdu 610065, China; 3 College of Physics and Information Engineering, Zhaotong University, Zhaotong 657000, China; shenyuncheng@ztu.edu.cn

**Keywords:** federated learning, data heterogeneity, catastrophic forgetting, knowledge distillation, self-adaption

## Abstract

As the development of the Internet of Things (IoT) continues, Federated Learning (FL) is gaining popularity as a distributed machine learning framework that does not compromise the data privacy of each participant. However, the data held by enterprises and factories in the IoT often have different distribution properties (Non-IID), leading to poor results in their federated learning. This problem causes clients to forget about global knowledge during their local training phase and then tends to slow convergence and degrades accuracy. In this work, we propose a method named FedRAD, which is based on relational knowledge distillation that further enhances the mining of high-quality global knowledge by local models from a higher-dimensional perspective during their local training phase to better retain global knowledge and avoid forgetting. At the same time, we devise an entropy-wise adaptive weights module (EWAW) to better regulate the proportion of loss in single-sample knowledge distillation versus relational knowledge distillation so that students can weigh losses based on predicted entropy and learn global knowledge more effectively. A series of experiments on CIFAR10 and CIFAR100 show that FedRAD has better performance in terms of convergence speed and classification accuracy compared to other advanced FL methods.

## 1. Introduction

With the growth of the Internet of Things and advances in Big Data-driven artificial intelligence, the network’s data are increasingly created by geographically distributed enterprise endpoints and IoT devices. The IoT in the context of Big Data is growing rapidly in the industrial sector. However, centralized aggregation of industrial Big Data to cloud servers leads to unaffordable transmission overheads and also violates the data privacy of each enterprise or client, which results in distributed databases consisting of multiple “data islands”. In light of the challenges posed by “data islands” in the development and application of the Internet of Things, Federated Learning (FL) [1] was first proposed in 2016 for collaborative learning with privacy constraints. It has been widely used in the IoT tasks such as smart cities [2], healthcare [3,4,5] and financial security [6]. Meanwhile, in the industrial Internet of Things, the use of federated learning to enable collaborative training of all parties while ensuring sensitive enterprise data is gradually becoming mainstream.

Although federated learning does not require centralized data aggregation to the cloud, however, there will be skewed distribution of data across enterprises during practical applications, which will lead to degradation of FL performance. For example, the data collected for mobile terminal input methods have different distributions for people with different operating habits. There are also many differences in the distribution of sensor acquisition data used by different plants to classify equipment faults or detect quality defects, both in terms of sensor type acquisition differences and fault type distribution differences. Wearable IoT sensor data for monitoring patient vital signs in the medical field are also heavily used for artificial intelligence learning to enable online diagnostics such as expert systems. However, the data collected by these edge IoT devices still suffer from inconsistencies in the distribution of features and labels. In summary, this huge challenge called Non-IID hinders the application of federated learning in IoT in the context of Big Data, and this work aims to propose a generalized method to address this challenge in federal learning for IoT applications.

**Figure 1:**
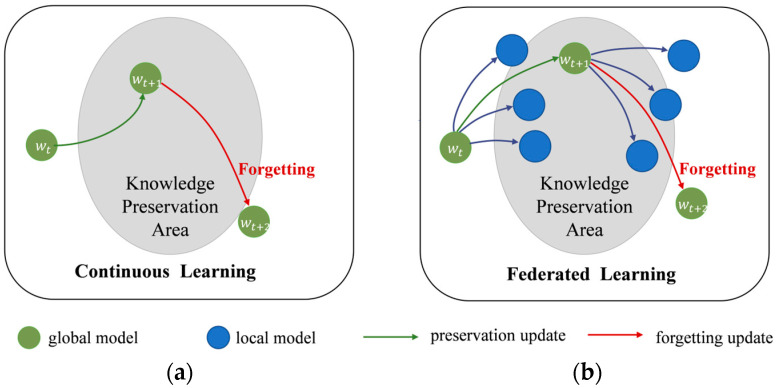
Catastrophic forgetting. (**a**) Forgetting in continuous learning; (**b**) forgetting in federated learning.

Similar to the problem in continuous learning [7], this variation among distributions causes each client to forget the global knowledge during their local updates, which in turn severely affects the performance and convergence in federated learning [8,9]. Figure 1 illustrates the catastrophic forgetting in continuous learning and federated learning. This phenomenon is referred to as “Client-drift” in [10]. FedProx [11] constrains the local updates by adding a regularization term to the local objective function to regulate its update direction towards the global objective. In recent years, knowledge distillation [12] is widely used for transferring knowledge between models to serve the purpose of compressing model size and improving model accuracy. In order to get a more robust global model, ref. [13] combines federated learning with knowledge distillation to fine-tune the global model after aggregation with an additional public dataset. Ref. [14] applies large model self-distillation on the server side to better maintain global knowledge. All these methods require an auxiliary public dataset for knowledge distillation, and FedGEMS even requires homogenous datasets for auxiliary data. Considering the existence of the forgetting phenomenon in federated learning, which is similar to continuous learning, especially in the case of data heterogeneity, this work attempts to introduce knowledge distillation in the local training phase, using collaborative distillation of global and local models to retain each other’s knowledge. Inspired by Relational KD [15], the high-dimensional relational knowledge naturally contained in a global model is distilled after each aggregation to achieve better performance in combination with single-sample knowledge. Relational knowledge distillation considers that “relationships” among knowledge are more representative of the teacher’s “knowledge” than separate representations. Similar to the view in linguistic structuralism [16], which focuses on structural relationships in symbolic systems, primary information is often located in structural relationships in the data embedding space rather than existing independently. Meanwhile, in order to weigh the effect of constraints on single-sample knowledge versus relational knowledge, this work introduces an adaptive coefficient module to dynamically adjust its constraints.

Inspired by above considerations, we propose a relational adaptive distillation paradigm called Relational Adaptive Distillation for Heterogeneous Federated Learning, abbreviated as FedRAD. The aggregated global model is downloaded by the selected clients during each round of communication and collaboratively distilled [17] with their own local models, transmitting both single-sample knowledge based on classical knowledge distillation and relational knowledge based on high-dimensional structural representations. This method can fully exploit the potential of knowledge distillation to exploit various types of knowledge in distributed data, which helps to motivate local models to learn higher dimensional knowledge representations from global models and minimize the forgetting phenomenon of local training in data heterogeneous scenarios. To better weigh the penalty focus of single-sample knowledge versus relational knowledge, we further propose an entropy-wise adaptive weight (EWAW) strategy to help local models adaptively control the impact of distillations based on the global model’s predictions on each data batch to prevent excessive transfer of negative knowledge. When the prediction of the global model is plausible, the local model learns the single-sample knowledge and relational knowledge in a balanced way. Otherwise, the local model focuses more on relational knowledge.

Our main contributions:

1. To address the phenomenon of drift and forgetting arising from local training of FL algorithms in IoT application deployment. This work incorporates an auxiliary constraint on loss in the local training phase in conjunction with relational knowledge distillation, enabling the local model to learn and retain global knowledge with a higher dimensional view to avoid forgetting.
2. To further improve model stability and robustness, this work proposes a robust local learning method named entropy-wise adaptive weights (EWAW), where the penalty weights between distillation losses are determined adaptively by the prediction entropy of global model on each local data model, which can further improve the model performance in each scenario by dynamically adjusting the loss weights.
3. By performing comprehensive experiments on CIFAR10 and CIFAR100, we validate that FedRAD has superior accuracy and convergence speed compared to FedAvg, FedProx and FedMD for scenarios with different levels of data heterogeneity and different percentages of active clients.

## 2. Related Work

### 2.1. Federated Learning

With the emergence of various data privacy protection requirements, secure multi-party computing [18,19] is commonly used in the past as the major method to resolve the conflict between data confidentiality and sharing in the IoT. However, its huge error accumulation and high computational cost in deep learning applications make it difficult for it to be competent for deep learning scenarios. In contrast, federated learning is widely used in deep learning as an emerging distributed learning paradigm. FedAvg [1] is a traditional classical federated learning paradigm, where the parameters or gradients of all local models are aggregated by the server to form a global model after some local updates are performed by each client, and the aggregation weights are proportional to the local data size. A key challenge of this classical paradigm is that the clients’ data are usually non-identically distributed (Non-IID). Many works have attempted to solve the Non-IID problem by improving the server aggregation phase or the local training phase. Refs. [20,21] start from a clustering perspective by assuming that there are differences in the similarity of data distributions among different clients, assigning similar clients into a cluster and implementing global model training within each cluster to reduce the impact of non-identical distributions. These methods are premised on the assumption of similarity in the distribution of client data, perform poorly in scenarios where the distribution of client data varies too much and fail to truly address the forgetting phenomenon that occurs during local training. Refs. [11,22,23] aim to improve the local training phase by adding a regularization term to local model as a constraint to adjust the deviation between local and global models and reduce the client drift phenomenon. Another technical route is to improve the server-side aggregation phase [24,25]. Unlike this route, this paper aims to preserve the global model knowledge in the local training phase, which belongs to improve the local training method.

### 2.2. Knowledge Distillation in FL

Knowledge distillation can transfer knowledge from large teacher models to small student models and is widely used for model compression [26,27] and collaborative learning among students to improve performance [17,28]. In order to address data heterogeneity, knowledge distillation applied to federated learning has proven to be an effective approach. Many works take aim at ensemble distillation, i.e., transferring knowledge to a global model as the student by aggregating client knowledge as the teacher. Ref. [29] use transfer learning on public datasets and ensemble distillation on the client side to improve model performance and reduce communication consumption. This is achieved by accomplishing knowledge transfer while exchanging only model predictions rather than model parameters. Ref. [13] combine federated learning with knowledge distillation to fine-tune the global model after aggregation with an additional public dataset in order to get a more robust global model. Ref. [14] improve on FedMD by holding a large model on the server side for self-distillation to better preserve global knowledge, which also avoids the forgetting of knowledge by the model. However, all these FL methods above require an additional public dataset similar to the client’s private datasets for knowledge distillation, and these carefully prepared public datasets are not always available. Some recent works attempt to extract knowledge without using additional public datasets: Ref. [30] aggregates and averages the logits in different classes transferred by each client on the server side for distribution as distillation knowledge in order to avoid reliance on public datasets. However, this method directly averages all logit of the same class, which tends to blur the knowledge across clients. Ref. [31] combine split learning to split the local model into a feature extraction network and a classification network, which use the intermediate features as inputs transmitted to the server-side classification network for knowledge distillation. This approach reduces the high computing power requirements for edge computing but performs poorly in the data heterogeneity scenario. Ref. [32] propose that the triangular upper bound of the federated learning objective function should be optimized especially in Non-IID scenarios, where both local training and knowledge distillation are used to lower the upper bound to improve performance. Refs. [33,34,35] use a pre-trained generator to generate pseudo data as a public dataset to assist in training, e.g., FedFTG trains a generator against the global model to generate difficult pseudo data in order to assist in training the global model to avoid forgetting. However, these methods of using a generator to add data require extremely high-quality generated samples, and often need a large computational cost to obtain high-quality samples. Ref. [36] perform knowledge distillation in the local training phase by broadcasting local data representations and the corresponding soft predictions, which is named “hyper-knowledge”. This method has some similarity to ours, without the need for generative model and public datasets, but is more concerned with balancing the performance of the local and global models.

In summary, to eliminate the dependence of the above knowledge distillation-based federated learning on public datasets and to fully exploit the potential of knowledge distillation to improve the performance of federated learning under data heterogeneity, this paper proposes FedRAD, which incorporates both relational knowledge and single-sample knowledge into the loss function in a collaborative distillation way between models, and adds adaptive weights based on prediction entropy to automatically control the penalty weights of the two distillation losses.

## 3. Methodology

**Figure 2:**
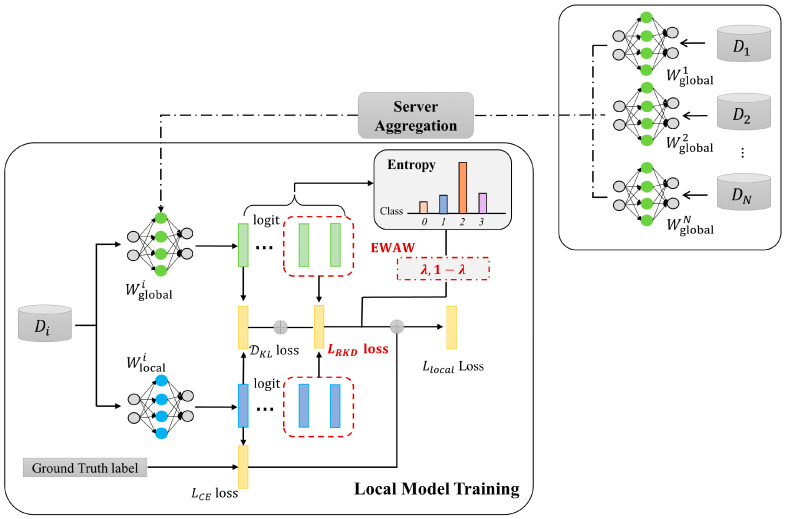
The general framework of FedRAD. In the local model training phase, after receiving global model, the selected client *i*’s model learns from local dataset $D_{i}\;$ by minimizing $L_{CE}$ loss. $D_{KL}$ loss and $L_{RKD}\;$ loss are utilized to transfer single sample knowledge and relational knowledge through collaborative distillation between local model and global model. The EWAW module calculates the entropy-wise weights which are used to adaptively determine the two distillation loss weights and makes the model more robust and reliable.

To address the inconsistency of learning and the consequent drift caused by the forgetting phenomenon in federated learning, this paper proposes a novel federated learning method called FedRAD from the perspective of expanding the dimension of knowledge distillation. Figure 2 visualizes the general framework of FedRAD. In detail, same as the classic federated learning framework, FedRAD consists of two main components: (1) server aggregation and (2) local model training. In each communication round, FedRAD randomly selects a group of clients and broadcasts the global model to them. Then, each selected client performs their own local model training, which is the main focus of FedRAD. After the local training phase, FedRAD performs the server aggregation. The server collects the selected local models and aggregates them as a new round global model by averaging the model weights for the next communication round.

### 3.1. Inconsistency in Federated Learning

**Figure 3:**
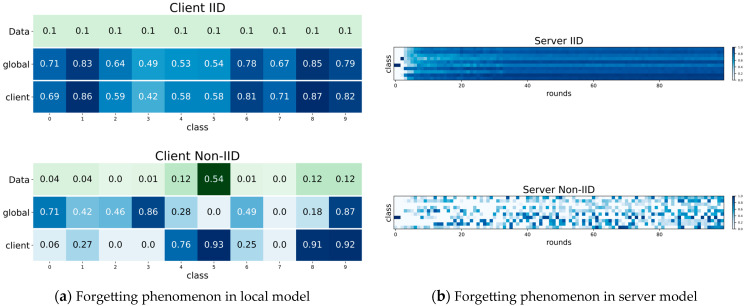
Forgetting phenomenon in federated learning: (**a**) confusion matrix of CIFAR10 under IID (data homogeneity) and Non-IID (data heterogeneity) scenarios which contains the data distribution status (the first row), the accuracy of the global model on each class before local training (the second row) and the accuracy of the global model on each class after local training (the third row), respectively; (**b**) IID and Non-IID scenarios of the global model accuracy per class on CIFAR10 with respect to the number of communication rounds.

To confirm the conjecture of incoherent learning due to forgetting in federation learning, we first built an image classification system using FedAvg as the federated learning framework. CIFAR10 (see details in Section 4.1.1) was selected as the dataset and it was classified into IID and Non-IID scenarios using the Dirichlet distribution. We trained the FedAvg for 20 communication rounds (avoiding the initial and ending training phases) and then verified its per-class accuracy. After that, we analyzed the per-class accuracy change of one client by training local models for a certain epoch. As shown in Figure 3a, the accuracy of the client model after local training in the Non-IID scenario is increased on certain classes which has more private data. However, the accuracy on the rest of classes is much smaller than the accuracy of global model it just received, implying that it forgot the received global knowledge. On the contrary, in the IID scenario, the global knowledge is preserved due to the homogeneous data distribution.

Then we further consider how the prediction of the global model changes as the number of communication rounds grows. The configuration of the experiment is the same as above except for the communication rounds. We also use CIFAR10 as the dataset and classify it into IID and Non-IID scenarios, respectively. As shown in Figure 3b, experiments on FedAvg for 100 communication rounds are conducted and record the prediction accuracy of global model for each class in each round. The results show that in the IID scenario, the global model learns very coherently and accumulates knowledge more uniformly. On the contrary, the prediction accuracy of global model for each class in different rounds in the Non-IID scenario is more variable, and the previous higher accuracy of a certain class is often accompanied by a subsequent decrease. The above analysis illustrates that similar to the forgetting phenomenon existing in continuous learning due to different data distributions, the forgetting of global knowledge also exists in federated learning under heterogeneous data scenarios.

### 3.2. Preliminary

The objective of this work is to train a classification model in a federated learning system without the need to train each client’s data centrally. This assumes that it contains N clients and 1 server and client n contains its private data $\;D_{n}≔\left\{x_{i}^{n},y_{i}\right\}$, where $x_{i}\;$ represents the *i*-th sample in local dataset and $y_{i}$ is its corresponding label, $y_{i}\in \{1,2,\ldots ,K\}$. Classical federated learning can be described as solving the following optimization problem:

(1) $$\underset{w}{min}L(w):=\sum_{n=1}^{N}\frac{\left|D_{n}\right|}{\left|D\right|}L_{n}(w),\;where\;L_{n}(w)=\frac{1}{\left|D_{n}\right|}\sum_{i=1}^{\left|D_{n}\right|}L_{CE}(w;x_{i},y_{i})$$

The global objective function L(w) is obtained by a weighted average of local objective functions from N clients, and the weights of clients are usually proportional to their private data amount $\left|D_{n}\right|$. At each communication round, the server distributes global model parameters aggregated in the previous round to the selected clients in order to solve Equation (1), and the clients perform local updates with their local objective function $L_{CE}$ on their respective local datasets $D_{n}$. Then server aggregates the updated parameters of the local models and forms a new global model.

However, this simple parameter-based aggregation approach does not perform well in the Non-IID scenario because the sample distribution is usually inconsistent across clients. The distribution fitted by each client’s local objective function is not consistent with the global objective function, which can easily lead to the forgetting phenomenon and, thus, the discarding of the global aggregation knowledge.

### 3.3. Codistillation in Local Training

During each communication round t, clients selected by the server get the global model from server side, while at the same time maintaining their own local models throughout the entire federated learning process. Both the local model’s loss and the global model’s loss during the local training phase are made up of cross-entropy loss and distillation loss, i.e., single-sample distillation loss and relational distillation loss. The local model is trained to gain single-sample distillation knowledge and relational distillation information from the global model, and the downloaded global model is trained in a similar manner to extract local model knowledge as well. This is accomplished through the process of collaborative distillation, which is performed by both sides. At the end of this iteration, each client will transmit the server their latest version of the global model. The server will then aggregate all of these versions into a new global model before beginning the process again. The loss of the local model and the global model are reformulated as follows:

(2) $$L_{local}=\alpha L_{CE}+(1-\alpha )[\lambda D_{KL}(p_{g}(x)∥p_{n}(x))+(1-\lambda )L_{RKD}]$$

(3) $$L_{global}=\alpha L_{CE}+(1-\alpha )[D_{KL}(p_{n}(x)∥p_{g}(x))+L_{RKD}]$$

where α is a hyperparameter which controls the ratio between the cross-entropy loss and the global distillation loss. It can be set to a high value in the initial training phase to fully utilize the knowledge from the local dataset. As the number of communication rounds increases, exponential decay is applied to α to fully exchange the knowledge between the local and global models, enhancing the effects of collaborative training. λ in the local loss is an adaptive weighting factor, which is used to automatically weigh the proportion of single-sample knowledge and relational knowledge in each batch from the perspective of prediction entropy; the detailed process is described in Section 3.4. The global model in the local side uses the local model to extract single-sample knowledge and relational knowledge in the same proportion so that it can be used in the subsequent round of aggregation.

The cross-entropy loss is calculated using the predicted value and the ground truth labels. Taking the local model n as an example, $z^{n},z^{g}$ represent the logit output of client’s local model and global model respectively, and the dataset of local model n is $D_{n}=\{x,y\},y\in \{1,2,...,K\}$. Then, the cross-entropy loss of the global model and local model during local training is defined as:

(4) $$L_{CE}=\sum_{x{\in D}_{n}}^{\;}-ylog[p(x)]$$

where predictions $p\left(x\right)=p_{g}(x)$ for the global model and $p\left(x\right)=p_{n}(x)$ for the local model respectively, where $p_{g}(x)$ and $p_{n}(x)$ are:

$$p_{g}(x)=\frac{\exp\left(z^{g}/T\right)}{\sum_{k=1}^{K}exp(z^{g,k}/T)},\;p_{n}(x)=\frac{\exp\left(z^{n}/T\right)}{\sum_{k=1}^{K}exp(z^{n,k}/T)}$$

where k represents the category and T represents the distillation temperature for controlling the effect of positive and negative category knowledge.

$D_{KL}$ is the Kullback–Leibler (KL) Divergence, which is used to measure the similarity of the distributions between the global model output and the local model output so that the global model and the local model could distill each other’s single-sample prediction knowledge:

(5) $$D_{KL}\left(p_{g}\left(x\right)∥p_{n}\left(x\right)\right)=\sum_{x\in D_{n}}-p_{g}\left(x\right)log\left[\frac{p_{g}\left(x\right)}{p_{n}\left(x\right)}\right]$$

(6) $$D_{KL}\left(p_{n}\left(x\right)∥p_{g}\left(x\right)\right)=\sum_{x\in D_{n}}-p_{n}\left(x\right)log\left[\frac{p_{n}\left(x\right)}{p_{g}\left(x\right)}\right]$$

### 3.4. Relational Distillation

High-dimensional spatial relationships between model predictions often consider the Euclidean distance of a binary versus the angular relationship of a triple. In the federated learning scenario, the processing capabilities of edge clients are limited, so it only takes into account mining the knowledge of distance relations between model predictions. This is because mining this knowledge is more efficient than mining other details. For a given pair of sample data $\left(x_{i},x_{j}\right)$ in each mini-batch, the distance relationship is measured by the Euclidean distance:

(7) $$\psi_{D}(z_{i},z_{j})=\frac{1}{\mu }{∥\;z_{i}-z_{j}\;∥}_{2},\mu =\frac{1}{\left|D^{2}\right|}\sum_{x_{i},x_{j}\in D,i\neq j}{∥\;z_{i}-z_{j}\;∥}_{2}$$

where D represents a batch of data and μ is the average distance of all sample predictions in one batch. In order take into consideration the relative distance that exists between the outputs, the magnitude of the distance between them has been adjusted using the normalization factor μ. The overall RKD loss is defined as follows:

(8) $$L_{\mathrm{RKD}}=\sum_{x_{i},x_{j}\in D_{i},i\neq j}l_{\delta }(\psi_{D}(z_{i}^{g},z_{j}^{g}),\psi_{D}(z_{i}^{n},z_{j}^{n}))$$

where $l_{\delta }$ is huber loss which combines MSE and MAE, reducing the high penalty tendency of MSE for outliers and the excessive gradient of MAE for small loss points, thus making it a more robust loss function.

### 3.5. Adaptive Weighting Coefficient

A sample $\left(x_{i},x_{j}\right)$ is passed through the downloaded global model to obtain the prediction output $p_{g}\left(x_{i}\right)$, and the outputs of M samples in each batch are used to calculate the mean value of their entropy to evaluate the prediction performance and confidence of the global model for that batch on the local data:

(9) $$H(p_{batch})=-\frac{1}{\left|batch\right|}\sum_{i=1}^{M}p_{g}\left(x_{i}\right)logp_{g}\left(x_{i}\right)$$

According to [37,38], a lower entropy represents a higher confidence level, and vice versa. The mapping function is therefore used to control its weighting coefficients, ultimately defined as:

(10) $$\lambda =\frac{\eta }{\left(e^{H(p_{batch})}+1\right)}$$

To map the results to the matching interval, the hyperparameter η is set to 1.6, which aims to map different entropy values to suitable weight intervals (see experiments in Section 4.3). This choice is made in order to ensure that the results are accurate. Due to this, the proportion of weights assigned to single-sample knowledge as opposed to relational knowledge for each batch is adaptively decided based on the level of entropy. In situations where the global knowledge of each round does not contain enough trustworthy information for accurately predicting the local data, the weight of the more reliable relational knowledge of the high-dimensional space is scaled up, and, on the contrary, the weight of the single-sample knowledge is appropriately decreased. This process occurs so that the global knowledge of each round can more accurately predict the local data.

Algorithm 1 shows a detailed description for FedRAD. In each round t, we first sample a subset based on fraction C of clients, then each client receives the global model and perform their client update phase in parallel for E local epochs. In this phase, the local model and global model in each of the selected clients are trained by collaborative distillation and are updated by minimizing Equations (2) and (3), respectively. And in the server update phase, the server aggregates the selected clients’ models by averaging the model weights. The above process continues cyclically until the communication round reaches the preset T.
**Algorithm 1:** FedRAD.**Input**: *T*: communication round; *N*: client number; *C*: the fraction of active clients in each round; $\{D_{i}{\}}_{i\in \{1,\ldots ,N\}}$: local datasets of clients; $p_{g}\left(x\right),p_{n}\left(x\right)$: prediction of global model and local model respectively;
 η: learning rate; *E*: local epochs **Output:** $w=\left[w_{1},\cdots ,w_{N}\right]\cup w_{g}$1.  Initialize model parameters *w*
2.  **Server update:**
3.  **for** each round
t∈{1,2,…,T}
**do**
4.    $S_{t}\leftarrow$ random subset (*C* fraction) of the *N* clients 
5.    **for** each clients
$i\in S_{t}$ in parallel **do**
6.      
$w_{g,i}^{t}\leftarrow$$\;\mathrm{Client}\;\mathrm{update}\left(i,w_{g}^{t}\right)$
7.    **end for**
8.    **Server Aggregation:** 
$w_{g}^{t+1}=\frac{1}{\left|S_{t}\right|}\sum_{i\in S_{t}}w_{g,i}^{t}$
9.   **end for**
10.    **Client update** 
$\left(i,w_{g}^{t}\right)$:
11.    **for** each
e∈{1,2,…,E}
**do**
12.    **for** batch
$b=\{x,y\}\in D_{i}$
**do**
13.      
$L_{local}=\alpha L_{CE}+(1-\alpha )[\lambda D_{KL}(p_{g}(x)∥p_{n}(x))+(1-\lambda )L_{RKD}]$   ▷ in Equation (2)
14.      $L_{global}=\alpha L_{CE}+(1-\alpha )[D_{KL}(p_{n}(x)∥p_{g}(x))+L_{RKD}]$        ▷ in Equation (3)
15.      $w_{i}^{t}\leftarrow w_{i}^{t}-\eta \nabla L_{local}$
16.      $w_{g,i}^{t}\leftarrow w_{g,i}^{t}-\eta \nabla L_{global}$
17.    **end for**
18.    **end for**

In detail, on the basis of local dataset training in Equation (2), i.e., minimizing the $L_{CE}$ loss, FedRAD incorporates single-sample knowledge and relational knowledge distillation into mutual learning between the local model and the distributed global model. By minimizing the $D_{KL}$ loss and $L_{RKD}$ loss, respectively, the global model and local model can more robustly retain each other’s knowledge in the output space. λ is the adaptive weighting coefficient which is automatically calculated by Equation (10) and can adaptively determine the two distillation loss weights in order to obtain a more robust and reliable model performance. 

## 4. Experiment

### 4.1. Implementation Details

#### 4.1.1. Datasets and Data Allocation

CIFAR10 and CIFAR100 are selected to serve as benchmark datasets for evaluating FedRAD and other baselines. CIFAR10/100 are 10/100 classification datasets, respectively. CIFAR10 consists of 50,000 training images and 10,000 test images in 10 classes, which have 5000 and 1000 images per class. CIFAR100 has the same total number of images as CIFAR10, but it has 100 classes. CIFAR100 has 500 training images and 100 testing images per class. All images of CIFAR10/100 are 3-channel color images (32 × 32). These two datasets have moderate classification difficulty, sufficient categories and a span of difficulty between them. It is also easy to divide the classes into different distributions and there is no potential structure to influence the experimental results. Therefore, these two datasets are widely used in federated learning. 

**Figure 4:**
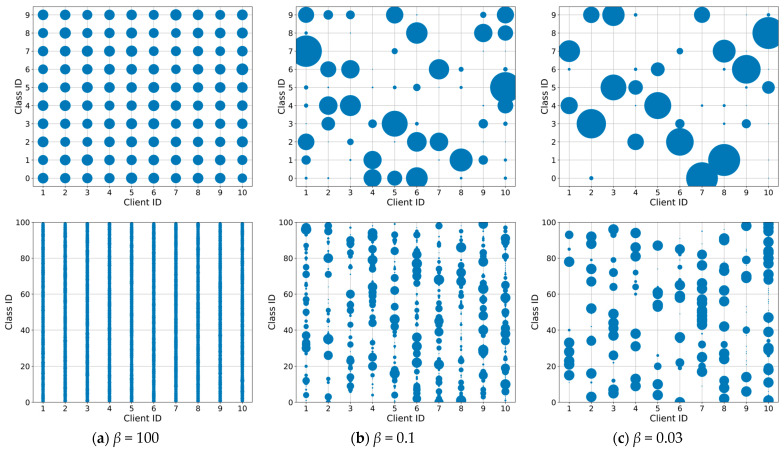
Classes allocated to each client at different Dirichlet distribution *β* for CIFAR-10 (on the top) and CIFAR100 (on the bottom) with 10 clients. The size of each dot reflects the magnitude of the samples number: (**a**) scenario of IID data; (**b**) scenario of Non-IID data in general; (**c**) scenario of Non-IID data in extreme case.

This work uses Dirichlet distribution to simulate different data distribution scenarios with unbalanced label classes, controlling the degree of Non-IID by a parameter  β. A smaller β indicates a greater degree of data heterogeneity, following some similar approaches [33,39]. We divide the Dirichlet division into 10 clients and simulate scenarios with IID and different degrees of Non-IID by choosing 100, 0.1 and 0.03 for β, which covers IID, normal Non-IID and extreme Non-IID scenarios. When beta is 100, each client has almost the same number of data classes, which simulates data homogeneity. When β is 0.03, however, it indicates that each client has almost only roughly two classes of data (using the CIFAR10 dataset). The relevant divisions are shown in Figure 4. 

#### 4.1.2. Baselines

FedRAD is designed to improve the forgetting phenomenon in federated learning with heterogeneous data, and we select some representative algorithms as baselines: FedAvg [1], FedProx [11] (regularization to improve model performance with heterogeneous data), FedMD [29] (knowledge distillation to improve model performance), FedDistill^+^ [34] (shares average logits through distillation) and FedGen (uses a generator to get expanded samples through clients’ prediction rules for knowledge distillation). For FedMD, as it requires a public dataset to be pre-trained for knowledge transfer, a portion of the CIFAR100 data is used as the public dataset in the CIFAR10 scenario. Similarly, a portion of the CIFAR10 data is used as the public dataset in the CIFAR100 scenario, and the accuracy median values of local models are illustrated. For FedDistill^+^, we also share the parameters of client models like FedAvg in order to make a fair comparison instead of just using FedDistill. The network of the generator used in FedGen is composed of two embedding layers (for inputs z and y, respectively) and two fully connected (FC) layers with LeakyReLU and BatchNorm layers between them, while the noise data z’s dimension d is 100 and 256 for CIFAR10 and CIFAR100, respectively.

#### 4.1.3. Models 

For CIFAR10 and CIFAR100, as well as for the different methods, ResNet18 [40] is chosen as the basic backbone. All models are implemented on Pytorch and run on an RTX3090 GPU. 

#### 4.1.4. Hyperparameters 

For all methods, the local training epoch is set to 5 and the number of communication rounds is set to 100, while the number of participating clients per round is set to 10 (with the activation ratio set to 1). For methods involving distillation, the distillation temperature T is set uniformly to 1. In local training phase, the batch size is set to 128 and the learning rate is set to 0.01 with an exponential decrease of 0.98. For FedProx, we adjust its proximal term in the range (0.001,0.01,0.1). The aggregation of knowledge in FedMD is performed using the averaging process used in the original paper.

### 4.2. Comparative Analysis of Accuracy and Efficiency

#### 4.2.1. Test Accuracy

**Figure 5:**
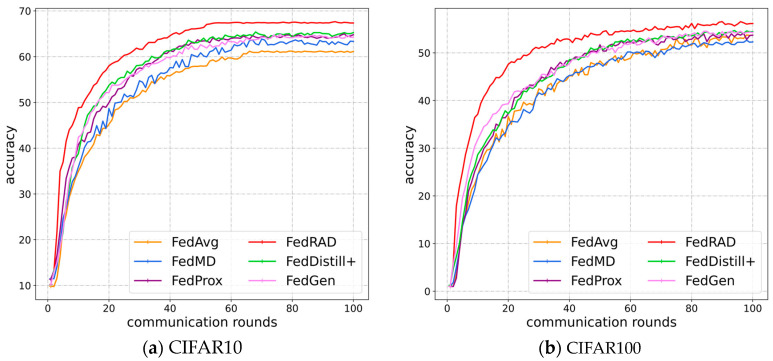
The learning curves of different FL methods on CIFAR-10 and CIFAR100 in Non-IID scenario (*β* = 0.1): (**a**) learning curves in CIFAR10; (**b**) learning curves in CIFAR100.

Table 1 shows the performance of different methods on CIFAR10 and CIFAR100 datasets, all experiments are repeated with 3 random seeds settings. As shown in Table 1, FedRAD outperforms the other methods in both homogeneous and heterogeneous data scenarios. Additionally, FedRAD outperforms FedProx, which uses proximal term to restrict the direction of updates, because it maintains both the global and local models locally and exchanges relational knowledge and single-sample knowledge between global and local models continuously, ensuring the model memory of global knowledge in a higher dimensional view. On the contrary, FedProx is more rigorous in restricting the update direction of the local model and less flexible in preserving global knowledge, which also has an impact on its convergence speed. On the other hand, although FedMD also uses knowledge distillation to exchange knowledge between local models, it performs poorly on heterogeneous data scenarios as it ignores high-dimensional relational knowledge and focuses only on single-sample knowledge. Although FedDistill^+^ combines parameter aggregation and knowledge distillation, the lack of dissemination of high-dimensional relational knowledge continues to make its performance weaker than FedRAD. Since FedGen uses a lightweight generator to add proxy data and it chooses to generate pseudo data on the feature level, its performance depends heavily on the generator’s quality. Meanwhile, the feature space of the data, although more compact than the input space, often does not carry enough knowledge to outperform other feature mining approaches, e.g., relational knowledge distillation. Figure 5 shows the learning curves of each method on CIFAR10 and CIFARI100 for the general data heterogeneity scenario (β=0.1). FedMD performs better than FedAvg at the beginning due to the addition of a pre-training process and knowledge transfer from the public dataset; however, the lack of knowledge mining at the convergence stage leads to a weaker performance than FedProx and a more prominent forgetting phenomenon. All the results show that FedRAD has a significant effect on the improvement of model accuracy in the data heterogeneity scenario.

#### 4.2.2. Communication Rounds

Table 2 shows the number of communication rounds consumed by different methods to achieve the target accuracy. Experiments are conducted at different degrees of Non-IID for CIFAR10 and CIFAR100. The results show that FedRAD takes the smallest communication rounds to achieve the proposed accuracy for different distribution scenarios in both datasets. FedRAD reaches the target accuracy faster than FedMD and FedProx. Compared to FedMD, FedRAD achieves better convergence speed due to a more complete retention of knowledge while having a resilient exchange of knowledge for learning through collaborative distillation in the local training phase, which can better overcome the forgetting phenomenon in federated learning. Unlike FedProx, which directly restricts updates to local models with global model parameters to alleviate forgetting, FedRAD uses knowledge distillation to constrain the update direction, while prediction entropy is evaluated for each batch to adaptively adjust the penalty weights between single-sample knowledge and relational knowledge. The impact of adaptive coefficients based on prediction entropy on the accuracy of model is further analyzed in Section 4.3.

### 4.3. Hyperparameters and Ablation Experiments

#### 4.3.1. Data Heterogeneity

**Figure 6:**
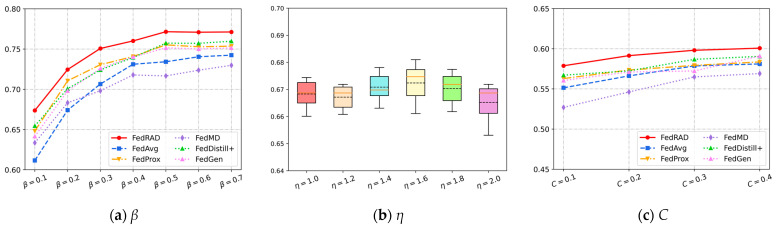
(**a**) Test accuracy with respect to data heterogeneity *β*; (**b**) test accuracy with respect to hyperparameter *η*; (**c**) test accuracy with respect to proportion of active clients C.

In order to assess the robustness of FedRAD under different degrees of data heterogeneity, the accuracy of different methods is tested under different Non-IID scenarios (adjusting for the hyperparameters of the Dirichlet distribution β). Figure 6a and Table 3 show the test accuracy of each federated learning method under different β in the CIFAR10 dataset. FedRAD achieves the best performance in various Non-IID distribution scenarios, outperforming other baselines. As the degree of data heterogeneity decreases (i.e., β increases), the accuracy of all methods tends to increase. FedProx performs similarly to FedAvg in some heterogeneous scenarios; one reason is that it simply imposes a regularization constraint on the local model based on the global model weights, rather than optimizing the local model with more specific constraints from a knowledge distillation perspective. It is also noted that FedRAD improves more than other methods in scenarios where the data are more heterogeneous, while FedMD does not improve significantly in this case, further validating the need for collaborative distillation to mine high-dimensional relational knowledge in the absence of single-sample knowledge. FedDistill^+^ aggregates the client model parameters and performs one-sample knowledge distillation for the global model, but it is also weaker than FedRAD in different distribution scenarios, which further illustrates the need for relational knowledge distillation in a Non-IID environment. FedGen’s performance in different Non-IID scenarios depends on the inductive bias it conveys to the clients, and the effect often depends on the quality of the feature space provided by the generator.

#### 4.3.2. Hyperparameters

As the key parameter that maps the prediction entropy to the corresponding coefficient interval, the hyperparameter η determines the penalty weight of single-sample distillation loss versus relational distillation loss under different sample predictions. To assess the effect of the key hyperparameter η on FedRAD, η is selected from (1.0,1.2,1.4,1.6,1.8,2.0) and experimented with in the CIFAR10 (β=0.1) case. When η=1, the entropy, even if small, i.e., when the prediction certainty is high, the single-sample knowledge distillation can still only obtain 1/2 of the weight coefficient. Figure 6b shows the accuracy of multiple experiments with different hyperparameter η choices in a box plot, which shows that FedRAD achieves the best performance with η=1.6, and the results also illustrate the robustness of FedRAD. As the value of η varies, the model accuracy is heavily concentrated between 0.66 and 0.68, which is not sensitive to the value of the hyperparameter η.

#### 4.3.3. Partial Client Participant

In order to evaluate the adaptability of FedRAD, experiments are conducted on CIFAR10 for different numbers of clients participating in each communication round. Specifically, the training data of CIFAR10 are assigned to 100 customers, and the proportion of active clients C is chosen as (0.1,0.2,0.3,0.4). Table 4 shows the test accuracy for each method with different proportions of active clients C. Figure 6c shows the test accuracy on different C. It can be seen in the figure that FedRAD achieves the best performance in each case. Additionally, when more clients participate in each communication round, each method’s performance improves.

#### 4.3.4. Ablation Study in FedRAD

Table 5 shows the accuracy of FedRAD after 100 rounds of training on CIFAR10 (β=0.1) with some key modules removed. Where $L_{RKD}$ stands for the rational knowledge distillation module, EWAW stands for entropy-wise adaptive weight and EDA stands for exponential decay of cross-entropy weight α. Removing the $L_{RKD}$ indicates that the collaborate distillation in Equations (2) and (3) only distill the single-sample knowledge, and the EWAW is naturally non-existent. In the early training phase, a large α allows models to initially learn knowledge from the dataset, and as the training rounds increase, α is decayed exponentially with EDA to expand the proportion of collaborative learning by knowledge distillation. It can be seen that the elimination of any part has some effect on the final test accuracy, especially when the adaptive coefficient adjustment EWAW is removed. This shows that it is crucial to evaluate the sample’s predictive entropy to adaptively adjust its penalty weight with respect to relational knowledge, to better propagate and retain more reliable knowledge and to reduce the forgetting of local models in federated learning. 

In summary, the series of experimental results show that FedRAD outperforms the benchmark method in terms of accuracy and convergence speed under different degrees of data heterogeneity and different client activity ratio scenarios, and has both strong robustness and adaptability. The results of the ablation experiments show that relational knowledge distillation in local training phase plays an important role and will influence the model performance, especially in Non-IID cases where the single prediction confidence is low. In addition, among the two modules, EWAW and EDA, EWAW improves FedRAD’s accuracy more significantly; meanwhile, EWAW is not sensitive to the value of hyperparameter η, and FedRAD achieves better performance in different parameter η selection scenarios.

## 5. Discussion

In the context of the rapid development of the Internet of Things and artificial intelligence driven by Big Data, more and more companies are seeking to use data from all parties for collaborative progress. Although the advent of federated learning has eliminated the need for enterprises or data terminals to upload large amounts of data to a central node, saving communication overhead and protecting data privacy, the data available to edge devices in the IoT often have large distribution differences, severely hindering the deployment and development of various IoT applications.

In this work, the proposed FedRAD makes full use of the knowledge distillation mining capability and achieves good performance in terms of model convergence speed and accuracy. For application scenarios with different degrees of data heterogeneity (β) and different proportions of active clients (*C*), FedRAD achieves a large advantage over other baselines, especially in extreme distribution heterogeneity cases. In terms of privacy protection, all training is done on the local side and no user data will be leaked. Overall, FedRAD is suitable for various scenarios of IoT application deployments due to its excellent performance in different scenarios, and it is able to solve the challenges caused by data heterogeneity such as intelligent transportation and industrial IoT collaboration.

The limitations of FedRAD are mainly in communication cost: FedRAD still uses the exchange of model network weights like FedAvg, which moderately increases the amount of communication compared to methods that only transfer predicted results. In addition, there is also a small increase in computational overhead due to the increased computation of relational knowledge distillation and prediction entropy for model training on the local side. Our future work will focus on how to further reduce communication overhead.

## 6. Conclusions

As a distributed learning paradigm that does not require centralized data and does not violate privacy, federated learning is prevalent in the deployment of Big Data-driven IoT applications. However, the data held by sensors and data collection terminals in IoT often have different distributions, which leads to degradation of FL performance. In order to address the phenomenon of drift and forgetting in FL, this paper proposes a generic method called FedRAD that enables models to learn knowledge from each other from a higher dimensional output space by incorporating single-sample knowledge and relational knowledge in a collaborative distillation approach to local training. Furthermore, an entropy-wise adaptive weights adjustment module (EWAW) is introduced to dynamically trade-off the constraint capacity between different distillation knowledge in different distribution scenarios. Experiments on two benchmark datasets, CIFAR10 and CIFAR100, demonstrate the well-adapted nature of FedRAD and its superiority in accuracy and convergence speed compared with the rest of the SOTA methods.

The future work will focus on how to reduce the communication overhead between IoT edge endpoints and server; one possible idea is to combine split learning to reduce the proportion of weights while making better use of knowledge distillation. Meanwhile, the method focuses on the local training phase, which is orthogonal to some of the methods used to improve the global aggregation phase, which could be combined with GAN in the future to improve the global model on the server side and further improve the forgetting phenomenon from the global side.

## Figures and Tables

**Table 1:** The top-1 test accuracy (%) of different FL methods on CIFAR10 and CIFAR100.

Method	Accuracy (%)					
	CIFAR10			CIFAR100		
	$\beta =100\left(IID\right)$	β=0.03	β=0.1	$\beta =100\left(IID\right)$	β=0.03	β=0.1
FedAvg	**86.54**	45.13	61.14	74.60	28.34	53.71
FedProx	85.69	49.33	64.81	**75.14**	32.78	53.77
FedMD	84.76	44.15	63.35	71.27	28.92	52.32
FedDistill^+^	86.34	49.21	65.46	75.11	32.55	54.49
FedGen	86.17	49.41	64.76	74.66	32.91	54.56
**FedRAD**	86.28	**52.23**	**67.37**	74.74	**34.88**	**56.12**

**Table 2:** Evaluation of different FL methods on CIFAR10 and CIFAR100 (*β* = 0.1) in terms of the number of communication rounds to reach target test accuracy.

Method	Communication Rounds			
	CIFAR10		CIFAR100	
	Acc=55%	Acc=60%	Acc=40%	Acc=50%
FedAvg	35	55	25	57
FedProx	26	36	21	43
FedMD	32	46	27	56
FedDistill^+^	25	35	22	43
FedGen	26	38	20	41
**FedRAD**	**17**	**24**	**11**	**26**

**Table 3:** The top-1 test accuracy with different data heterogeneity *β* on CIFAR10.

Method	Accuracy (%)						
	β=0.1	β=0.2	β=0.3	β=0.4	β=0.5	β=0.6	β=0.7
FedAvg	61.16	67.41	70.33	73.11	73.42	74.04	74.24
FedProx	64.81	71.21	72.85	74.07	75.52	75.28	75.36
FedMD	63.35	68.32	69.81	71.78	71.66	72.37	72.98
FedDistill^+^	65.46	70.07	72.41	73.95	75.73	75.71	75.97
FedGen	64.16	69.81	72.58	74.01	75.15	75.03	75.16
**FedRAD**	**67.37**	**72.65**	**75.07**	**76.18**	**77.25**	**77.18**	**77.21**

**Table 4:** The top-1 test accuracy with different proportion of active clients on CIFAR10 (*β* = 0.1).

Method	Accuracy (%)			
	C=0.1	C=0.2	C=0.3	C=0.4
FedAvg	55.14	56.62	57.86	58.11
FedProx	56.29	57.35	57.93	58.36
FedMD	52.70	54.62	56.49	56.91
FedDistill^+^	56.71	57.24	58.68	59.05
FedGen	56.05	57.12	57.23	59.09
FedRAD	**57.88**	**59.14**	**59.81**	**60.07**

**Table 5:** Impact of each module in FedRAD.

	Method	Accuracy (%)
Baseline	FedRAD	67.37
Module	$\mathrm{no}\;L_{RKD}$	64.45
	no EWAW	66.09
	no EDA	66.74
	no EWAW + EDA	65.71

## Data Availability

Not applicable.

## References

[1] McMahan B., Moore E., Ramage D., Hampson S., y Arcas B.A. Communication-Efficient Learning of Deep Networks from Decentralized Data. Proceedings of the 20th International Conference on Artificial Intelligence and Statistics, AISTATS 2017.

[2] Zheng Z., Zhou Y., Sun Y., Wang Z., Liu B., Li K. (2022). Applications of Federated Learning in Smart Cities: Recent Advances, Taxonomy, and Open Challenges. Connect. Sci..

[3] Liu Q., Chen C., Qin J., Dou Q., Heng P.-A. FedDG: Federated Domain Generalization on Medical Image Segmentation via Episodic Learning in Continuous Frequency Space. Proceedings of the IEEE Conference on Computer Vision and Pattern Recognition, CVPR 2021.

[4] Vaid A., Jaladanki S., Xu J., Teng S., Kumar A., Lee S., Somani S., Paranjpe I., Freitas J., Wanyan B. (2021). Federated Learning of Electronic Health Records to Improve Mortality Prediction in Hospitalized Patients with COVID-19: Machine Learning Approach. JMIR Med. Inform..

[5] Zhao L., Huang J. (2023). A Distribution Information Sharing Federated Learning Approach for Medical Image Data. Complex Intell. Syst..

[6] Byrd D., Polychroniadou A. Differentially Private Secure Multi-Party Computation for Federated Learning in Financial Applications. Proceedings of the ICAIF ’20: The First ACM International Conference on AI in Finance.

[7] Ring M.B., Thrun S., Pratt L.Y. (1998). Child: A First Step Towards Continual Learning. Learning to Learn.

[8] Zhao Y., Li M., Lai L., Suda N., Civin D., Chandra V. (2018). Federated Learning with Non-IID Data. arXiv.

[9] Li X., Huang K., Yang W., Wang S., Zhang Z. On the Convergence of FedAvg on Non-IID Data. Proceedings of the 8th International Conference on Learning Representations, ICLR 2020.

[10] Karimireddy S.P., Kale S., Mohri M., Reddi S.J., Stich S.U., Suresh A.T. SCAFFOLD: Stochastic Controlled Averaging for Federated Learning. Proceedings of the 37th International Conference on Machine Learning, ICML 2020.

[11] Li T., Sahu A.K., Zaheer M., Sanjabi M., Talwalkar A., Smith V. Federated Optimization in Heterogeneous Networks. Proceedings of the Machine Learning and Systems 2020, MLSys 2020.

[12] Hinton G.E., Vinyals O., Dean J. (2015). Distilling the Knowledge in a Neural Network. arXiv.

[13] Lin T., Kong L., Stich S.U., Jaggi M. Ensemble Distillation for Robust Model Fusion in Federated Learning. Proceedings of the Advances in Neural Information Processing Systems 33: Annual Conference on Neural Information Processing Systems 2020, NeurIPS 2020.

[14] Cheng S., Wu J., Xiao Y., Liu Y. (2021). FedGEMS: Federated Learning of Larger Server Models via Selective Knowledge Fusion. arXiv.

[15] Park W., Kim D., Lu Y., Cho M. Relational Knowledge Distillation. Proceedings of the IEEE Conference on Computer Vision and Pattern Recognition, CVPR 2019.

[16] Matthews P. (2001). A Short History of Structural Linguistics.

[17] Anil R., Pereyra G., Passos A., Ormándi R., Dahl G.E., Hinton G.E. Large Scale Distributed Neural Network Training through Online Distillation. Proceedings of the 6th International Conference on Learning Representations, ICLR 2018.

[18] Yao A.C.-C. Protocols for Secure Computations (Extended Abstract). Proceedings of the 23rd Annual Symposium on Foundations of Computer Science.

[19] Yao A.C.-C. How to Generate and Exchange Secrets (Extended Abstract). Proceedings of the 27th Annual Symposium on Foundations of Computer Science.

[20] Sattler F., Müller K.-R., Samek W. (2021). Clustered Federated Learning: Model-Agnostic Distributed Multitask Optimization Under Privacy Constraints. IEEE Trans. Neural Netw. Learn. Syst..

[21] Ghosh A., Chung J., Yin D., Ramchandran K., Larochelle H., Ranzato M., Hadsell R., Balcan M.F., Lin H. (2020). An Efficient Framework for Clustered Federated Learning. Advances in Neural Information Processing Systems.

[22] Hanzely F., Richtárik P. (2020). Federated Learning of a Mixture of Global and Local Models. arXiv.

[23] Dinh C.T., Tran N.H., Nguyen T.D. Personalized Federated Learning with Moreau Envelopes. Proceedings of the Advances in Neural Information Processing Systems 33: Annual Conference on Neural Information Processing Systems 2020, NeurIPS 2020.

[24] Chen H.-Y., Chao W.-L. FedBE: Making Bayesian Model Ensemble Applicable to Federated Learning. Proceedings of the 9th International Conference on Learning Representations, ICLR 2021.

[25] Wang H., Yurochkin M., Sun Y., Papailiopoulos D.S., Khazaeni Y. Federated Learning with Matched Averaging. Proceedings of the 8th International Conference on Learning Representations, ICLR 2020.

[26] Wang W., Wei F., Dong L., Bao H., Yang N., Zhou M. MiniLM: Deep Self-Attention Distillation for Task-Agnostic Compression of Pre-Trained Transformers. Proceedings of the Advances in Neural Information Processing Systems 33: Annual Conference on Neural Information Processing Systems 2020, NeurIPS 2020.

[27] Sun S., Cheng Y., Gan Z., Liu J., Inui K., Jiang J., Ng V., Wan X. (2019). Patient Knowledge Distillation for BERT Model Compression. Proceedings of the 2019 Conference on Empirical Methods in Natural Language Processing and the 9th International Joint Conference on Natural Language Processing, EMNLP-IJCNLP 2019.

[28] Zhang Y., Xiang T., Hospedales T.M., Lu H. Deep Mutual Learning. Proceedings of the 2018 IEEE Conference on Computer Vision and Pattern Recognition, CVPR 2018.

[29] Li D., Wang J. (2019). FedMD: Heterogenous Federated Learning via Model Distillation. arXiv.

[30] Jeong E., Oh S., Kim H., Park J., Bennis M., Kim S.-L. (2018). Communication-Efficient On-Device Machine Learning: Federated Distillation and Augmentation under Non-IID Private Data. arXiv.

[31] He C., Annavaram M., Avestimehr S. Group Knowledge Transfer: Federated Learning of Large CNNs at the Edge. Proceedings of the Advances in Neural Information Processing Systems 33: Annual Conference on Neural Information Processing Systems 2020, NeurIPS 2020.

[32] Li X., Chen B., Lu W. (2023). FedDKD: Federated Learning with Decentralized Knowledge Distillation. Appl. Intell..

[33] Zhang L., Shen L., Ding L., Tao D., Duan L.-Y. Fine-Tuning Global Model via Data-Free Knowledge Distillation for Non-IID Federated Learning. Proceedings of the IEEE/CVF Conference on Computer Vision and Pattern Recognition, CVPR 2022.

[34] Zhu Z., Hong J., Zhou J. Data-Free Knowledge Distillation for Heterogeneous Federated Learning. Proceedings of the 38th International Conference on Machine Learning, ICML 2021.

[35] Zhang L., Wu D., Yuan X. FedZKT: Zero-Shot Knowledge Transfer towards Resource-Constrained Federated Learning with Heterogeneous On-Device Models. Proceedings of the 42nd IEEE International Conference on Distributed Computing Systems, ICDCS 2022.

[36] Chen H., Wang C., Vikalo H. The Best of Both Worlds: Accurate Global and Personalized Models through Federated Learning with Data-Free Hyper-Knowledge Distillation. Proceedings of the Eleventh International Conference on Learning Representations, ICLR 2023.

[37] Pereyra G., Tucker G., Chorowski J., Kaiser L., Hinton G.E. Regularizing Neural Networks by Penalizing Confident Output Distributions. Proceedings of the 5th International Conference on Learning Representations, ICLR 2017.

[38] Szegedy C., Vanhoucke V., Ioffe S., Shlens J., Wojna Z. Rethinking the Inception Architecture for Computer Vision. Proceedings of the 2016 IEEE Conference on Computer Vision and Pattern Recognition, CVPR 2016.

[39] Sattler F., Korjakow T., Rischke R., Samek W. (2021). FEDAUX: Leveraging Unlabeled Auxiliary Data in Federated Learning. IEEE Trans. Neural Netw. Learn. Syst..

[40] He K., Zhang X., Ren S., Sun J. Deep Residual Learning for Image Recognition. Proceedings of the 2016 IEEE Conference on Computer Vision and Pattern Recognition, CVPR 2016.

